# Application of integrated yoga therapy to increase imitation skills in children with autism spectrum disorder

**DOI:** 10.4103/0973-6131.66775

**Published:** 2010

**Authors:** Shantha Radhakrishna

**Affiliations:** Sri. Ganapathi Sachchidananda (SGS) Vagdevi Centre for the Rehabilitation of Communication Impaired, Bangalore, Karnataka, India

**Keywords:** Autism, Yoga, Imitation

## Abstract

**Background/Aim:**

Children with autism exhibit significant deficits in imitation skills, which impede the acquisition of more complex behavior and socialization. Imitation is often targeted early in intervention plans and continues to be addressed throughout the child’s treatment. The use of integrated approach to yoga therapy (IAYT) as a complementary therapy for children diagnosed with autism spectrum disorder (ASD) is rarely reported and little is known on the effectiveness of such therapies. This study investigated IAYT as a treatment method with children with ASD to increase imitative skills.

**Materials and Methods:**

Parents and six children with ASD participated in a 10-month program of 5-weekly sessions and regular practice at home. Pre, mid and post treatment assessments included observers and parent ratings of children’s imitation skills in tasks related to imitation skills such as gross motor actions, vocalization, complex imitation, oral facial movements and imitating breathing exercises.

**Results:**

Improvement in children’s imitation skills especially pointing to body, postural and oral facial movements. Parents reported change in the play pattern of these children with toys, peers and objects at home.

**Conclusions:**

This study indicates that IAYT may offer benefits as an effective tool to increase imitation, cognitive skills and social-communicative behaviors in children with ASD. In addition, children exhibited increased skills in eye contact, sitting tolerance, non-verbal communication and receptive skills to verbal commands related to spatial relationship.

## INTRODUCTION

The ability to understand another person’s action and, if needed, imitate that action is a core component of human social behavior. Imitation skills can be observed as early as infancy. In typical infants, imitation emerges early in development and plays a crucial role in the development of cognitive, social, communication and other behaviors such as language, play and joint attention.[1]

Early imitation is a non-verbal means of information processing. In normal development, the baby is not taught imitation as such; only in the second half of the year parents begin to teach imitation like waving bye-bye, etc. Typical children with autism spectrum disorder (ASD) fail to demonstrate these skills. The more social the imitation is, the harder it is to master. In the order of difficulty, spontaneous object use is least difficult, motor object imitation difficult and body imitation most difficult.[2] Motor imitation is a complex developmental phenomenon that serves important cognitive and social functions. At a social level, it represents earliest forms of reciprocal interactions between infant and the mother.

There is a growing body of literature demonstrating that children with autism have specific deficits in imitating action on objects, body movements, vocalization, gesture, functional objectives and facial expression. Most researchers recognize imitation as a central deficit in children with autism[3 4] and a lack of imitative play is one of the diagnostic criteria for the disability.[5]

Imitation is defined as the reproduction of a model’s action in topography and function for the new actions only. Charmil and Baren-Cohen,[6] Dawson and Adams[7] and De Myer[8] were among the first to investigate imitation skills in autism. In their experiment, 12 children with autism and early childhood schizophrenia were compared to a controlled group of children with mental retardation. The groups were evaluated on a variety of body movements and object manipulation. Children with autism exhibited significantly less imitation skill and had particular difficulty with gestural imitation. Many studies supported these findings. Heimann, Ullstadius, Danigren and Gilberg[9] also found that motor tasks were the least frequently imitated categories in children with autism. Many more studies confirmed the above findings of a relative deficit in motor imitation in children with autism.[10]

Imitation research on children with autism has focused primarily on the form of imitation (i.e. gestural, object, facial, vocal). Cognitive developmental research on imitation in autism generally used Piagetian models of sensory motor development and compared children with autism to mental age matched peers on a series of sensory motor tasks. In behavioral research of imitation in children with autism, emphasis is often placed on factors influencing skill acquisition including teaching factors such as presentation mode and model type. Independent variables evaluated in behavioral analytic literature typically include response class generalization and peer modeling. De Myer’s[8] initial research generated many studies supportive of the general findings of imitation deficits in autism. Findings of deficits in imitation skills have significant implications for the intervention approaches given the critical nature of imitation to one’s ability to learn from the environment.

Treatment for autism based on either behavioral or cognitive developmental models emphasizes on developing imitation skills in young children with autism. The methods and treatments used remain to be empirically validated.

Many behavioral treatment approaches focusing on imitation are in use in treating children with autism. In discrete trial training (DTT), a target behavior or skill is broken into component parts and repeatedly practiced with prompting and fading the prompting until the skills are mastered.

The applied behavioral analysis (ABA) also includes teaching imitation skills in a “command/prompt method” where a teacher provides a prompt or command for the autistic student to initiate and if the student achieves the desired behavior, there are rewards and if not, there are repeats of the command/prompt and a repeat for the student to produce the expected behavior. The desired behavior is then reinforced and the student can repeat the expected behavior in the classroom.

There are many behavioral treatment approaches such as language training behavior, natural language procedure, incidental teaching, pivotal learning and errorless learning. All these basic procedures use ABA principles such as stimulus control, prompts, modeling, shaping and reinforcement to teach imitation skills. All these procedures consider imitation skills to be essential to new learning. Imitation skills are typically among the first to be taught in many of these programs because they are often considered being pre-requisite abilities for learning other skills, e.g. motor imitation (clapping, running, walking on toes and jumping). Once basic imitation skills are established, they can be used as building blocks for complex tasks.

A pilot study by Radhakrishna[11] suggests that integrated approach to yoga therapy (IAYT) can specifically increase imitation skills, an essential pre-requisite for learning. It also demonstrated changes in non-verbal communication, self-esteem, emotional bonding, focus, tolerance to touch, proximity and sharing of attention.

The study reported here started with the premise that as clinicians, we need to develop intervention approaches that are derived from a number of theoretical understandings of autism.

The IAYT approach is based on the philosophy that the child is perfect and whole, and that the child and therapist are both unlimited in their abilities to teach. Supporting these beliefs is empirically sound therapy based on yoga philosophy and practice to help the child to reach his/her highest potential for a quality life.

## MATERIALS AND METHODS

### Subject selection

This study adopted a case study approach. The IAYT program was publicized through workshops conducted at various national institutes and centers and schools for ASD children. Six children admitted to SGS Vagdevi Integrated School were matched for age, sex, IQ and socioeconomic/educational background of parents. Children who were already diagnosed with ASD by leading institutions of Bangalore, India, were selected for the study. Diagnosis was cross-validated by the author using DSM-IV-TR[5] criteria. The Childhood Rating Scale[12] was also used to determine autism severity. All the children demonstrated mild to moderate range of autism.

Data are given in Table 1.

**Table 1:** Demographic data

No.	Age	Sex	IQ	SEB	EB
6	8–14 years	M/F = 5/1	70 and above	Middle class	Graduates

SEB = Socio-economic background (minimum Rs. 8000); EB = Educational background (graduate mothers)

Written consent to participate in the study was obtained from the parents.

### Imitation test battery

The tasks given in Table 2 were developed for this study based on previous pilot study experience by the researcher who is a speech-language/yoga therapist by profession.

**Table 2:** Target imitation skills

Imitating gross motor actions	Imitating vocalizatione	Complex imitation	Imitating oral facial movements	Breathing exercises
Running Walking Jumping Walking on toes	Imitating sounds (A, E, U, OM) Imitating words Imitating phrases	Simple asanas Imitating sequence actions	Lips, tongue and jaw exercises	Blowing exercises In and out breathing Sectional breathing

### Assessment procedure

Special educators and parents contributed to a range of data collection procedure through questionnaire and observers’ comments and interviews. Assessment was conducted at 3 points. Pre (1–12 sessions), mid (60th, 80th and 100th sessions) and post (180th, 181st and 182nd sessions).

### Child assessment measures

Perceived outcomes of IAYT for the child were measured at the mid and end points of the program. Parents completed a short questionnaire to see whether IAYT has made any change on the five targeted areas of behavior. A simple 3-point rating scale was used (based on researcher’s pilot study[11]) to obtain information on the level of benefit (0 = rarely imitates, 1 = occasionally imitates, 2 = consistently imitates). Three trained observers completed the assessment. Responses were scored on a 3-point scale; a “2” was recorded if the child produced exact imitation, a “1” was recorded if the child produced an occasional imitation and a “0” was recorded if the child rarely imitates or imitation absent. Inter-rater reliability was established prior to scoring and maintained throughout the study.

### Yoga intervention

Yoga therapy was then introduced five times a week 45 minutes daily for 10 months. Mother accompanied the child during all these sessions. These sessions took place in an open green, serene spiritual atmosphere overlooking an ashram and temple. Children used their own mats and marked their own boundary of operation. Yoga asanas (postures) and pranayama (breathing exercises) adopted in this study were specially selected to address issues related to imitation difficulties with ASDs.

Exercises adopted during IAYT are listed in Table 3.

**Table 3:** Yoga intervention

Warm-up asanas	Strengthening asanas	Release of tension asanas	Calming asanas	Breathing asanas
Jogging Bending exercises Twisting	Trikonasana Parshavakonasana Veerabhadrasana	Neck exercises Back bending exercises Relaxation exercises	Sukhasana Shavasana	Blowing exercises in and out breathing sectional breathing

The sequence consisted of “warm-up asanas, strengthening asanas, release of tension asanas, calming asanas and breathing asanas”. Yogasanas selected initially were physically less demanding. During warm-up asanas, if the child did not imitate the therapist, the attending adult physically guided the child to complete the task. The child slowly learned that she/he is expected to imitate the model. It also provided a motor plan to complete more asanas.

## RESULTS AND DISCUSSION

First the children’s baseline data are described in relation to imitation skills. Second, changes in various parameters at mid and post therapy phase are discussed. In the third section, changes in the related behavior, namely communication, social relationships and behavioral perseveration, are elaborated. The initial interviews with parents and staff carried out to gain an insight into the current imitation behavior are summarized.

Relative absence of imitation was not of immediate concern to parents as their knowledge of the importance of imitation and its impact on development was limited. From the parents’ perceptive, lack of any form of communication, not playing with other children, hyperactivity was something they found particularly difficult to cope with.

**Figure 1:**
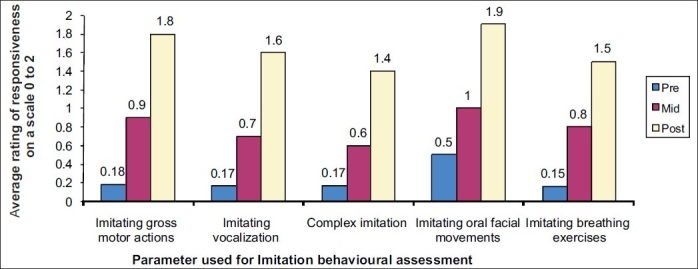
Graphic representation of observed improvement in selected imitation behavioral traits

The baseline, mid and post therapy data are summarized in Figure 1.

### Summary of imitation behaviors

At the start of the study, children in this sample

- never imitate gross motor actions (could not imitate the model’s actions of pointing to body parts)
- rarely imitate vocalization
- never imitate two phase complex movements
- rarely imitate oral facial movements
- never imitate adult breathing in and out model

All the six children performed poorly in imitating breathing exercises. They could not perform even simple blowing exercises. This may be directly related to their poor vocalization and expressive speech. They had difficulty imitating two or more sequence imitations, initiate vocalization and imitating gross motor actions. Children were clumsy with their movements, were poorly coordinated and could not run, jump, hop and walk on toes. Four out of six children occasionally imitated oral facial movements like protruding, elevating tongue and puckering lips. Generally, this supports previous research findings that children underperformed on various imitation tasks such as gross motor actions, vocalization, complex imitation, oral facial imitation and imitating breathing exercises.[10] Complex motor tasks were the least frequently imitated category compared to oral facial movement imitation. Children had more difficulty on tasks with multiple components than task involving one action. Attempts by the therapist to involve the child to sit in vajrasana (folding both legs backward and sitting on the heel) initially resulted in the child losing interest in therapy program and running away. No attempts were made to force the children on the therapy mat, but slowly they joined the group voluntarily.

During the mid assessment period, there was a significant change in imitating gross motor actions, oral facial movements and performing breathing exercises, but little change was seen in imitating complex imitation and vocalization.

During last few sessions, significant changes in the imitation skills related to all the five parameters and also changes in communication, functional object use, language, play and joint attention were seen. Pattern of eye contacts steadily improved. Children started focusing on the yoga therapist as she gave counts with drumbeat. Initially, mothers manually guided the children to imitate the movement. Slowly, manual manipulation decreased and children started imitating complex motor movements spontaneously. It is possible that a gentle touch or pressure gave them a different experience and they started perceiving changing dynamics and became interested in therapy. Consequently, children started to display early shared attention behaviors such as looking at the peers, making eye contact with the therapist and offering no resistance to the therapist.

In addition to these behaviors, an increase in facial expression (pain and pleasure), vocalizations and gazing at peers suggested an emerging understanding that sharing an activity could be an enjoyable experience.

As the therapy progressed, increase in imitation skills was noticed in imitating familiar and learnt movement. Children started looking at peer model, resulting in higher levels of generalization and maintenance of learnt imitation behavior. This supports the study by Carr and Darey[13] using different types of models (peer and adult) and suggesting that peer model would help in better generalization and maintenance of the learnt skill. Close physical proximity of the mother and prompting of a specific behavior by the mother may be a contributing factor for higher generalization.

All the six children started to indicate their preferences for asana, e.g. Shavasana, Parvathasana. They progressed from the early resistance to passive tolerance to active participation and enjoying the therapy sessions. Over the course of yoga therapy, children started to trust, share, initiate and reciprocate and thus the barrier to communication of carrying the label of being “autistic” is broken. By the end of 183rd session, all six children engaged in 30–45 minutes of yoga therapy. During this period, they all displayed increased intention to remain in close proximity with the therapist and participated in performing most of the asanas and breathing exercises.

All the six children showed increased vocal imitation skills by imitating vowels “a, e, i, o, u” and “OM”. This increased vocal imitation may be due to the verbal behavior approach adopted by the yoga therapist who is also a Speech-Language-Pathologist (SLP). Verbal behavior approach to teaching language to children with autism emphasizes teaching language units in its functional components such as manding (to alter one’s environment), tacting (to respond to sensory stimuli) and intraverbals (verbal behavior in response to another person’s verbal behavior). Imitation was used throughout the teaching of mands, tacts and intraverbals. Changes in social interaction were seen. Children started greeting the therapist with “namasthe” (with folded hands) and verbalized “om shanthi” (let there be peace) at the end of the therapy session.

Children engaged in increased play interaction during yoga therapy sessions. Children who display increased imitation skills during yoga therapy transferred these responses into play situation whenever they engaged in symbolic play. Final interviews with parents and staff were carried out to assess whether the child’s imitation skill has changed over the course of the study. Parents reported that their children indicated basic needs using gestures, interacting with other children during play situation and increased sitting tolerance for an activity.

To conclude, this is the first scientific study in India investigating the effect of IAYT to increase imitation skills and also related language, social and cognitive skills. This study aimed to investigate IAYT as a family-oriented treatment alongside any conventional treatment received by the children. The pilot study provides initial evidence of the benefits of IAYT in alleviating the behavioral symptoms of children diagnosed with ASD, confirmed through parents’ and teachers’ report and children’s own behavior. Future directions in IAYT research would be well served by larger studies that involve teachers as well as parents, followed by follow-up studies. Rigorously controlled clinical trials on larger and more homogeneous population would be needed to provide the necessary rigor to assess the relative effect of IAYT as an alternative or complementary treatment to increase imitation skills in children with ASD. However, the indications are that IAYT may offer families an effective management tool for family-oriented treatment of childhood ASD.
